# IL-1β may be an indicator of peritoneal deterioration after healing of peritoneal dialysis-associated peritonitis

**DOI:** 10.1186/s12882-023-03431-8

**Published:** 2023-12-19

**Authors:** Akira Hirano, Hiroyuki Kadoya, Yu Yamanouchi, Seiji Kishi, Tamaki Sasaki, Naoki Kashihara

**Affiliations:** Department of Nephrology and Hypertension, Kawasaki Medical School, Kurashiki, Okayama 701-0192 Japan

**Keywords:** Chronic inflammation, IL-1β, Inflammasome, Peritoneal dialysis, Peritonitis

## Abstract

**Background:**

Peritoneal dialysis (PD) is an essential lifesaving treatment for end-stage renal disease. However, PD therapy is limited by peritoneal inflammation, which leads to peritoneal membrane failure because of progressive peritoneal deterioration. Peritonitis is the most common complication in patients undergoing PD. Thus, elucidating the mechanism of chronic peritoneal inflammation after PD-associated peritonitis is an urgent issue for patients undergoing PD. This first case report suggests that an increased interleukin-1β (IL-1β) expression in the peritoneal dialysate after healing of peritonitis can contribute to peritoneal deterioration.

**Case presentation:**

A 64-year-old woman was diagnosed with diabetes mellitus 10 years ago and had been started on PD for end-stage renal disease. One day, the patient developed PD-associated acute peritonitis and was admitted to our hospital for treatment. Thus, treatment with antimicrobial agents was initiated for PD-associated peritonitis. Dialysate turbidity gradually disappeared after treatment with antimicrobial agents, and the number of cells in the PD fluid decreased. After 2 weeks of antimicrobial therapy, peritonitis was clinically cured, and the patient was discharged. Thereafter, the patient did not develop peritonitis; however, residual renal function tended to decline, and peritoneal function also decreased in a relatively short period. We evaluated pro-inflammatory cytokine levels before and after PD-associated peritonitis; interestingly, the levels of IL-1β remained high in the PD fluid, even after remission of bacterial peritonitis. In addition, it correlated with decreased peritoneal function.

**Conclusions:**

This case suggests that inflammasome-derived pro-inflammatory cytokines may contribute to chronic inflammation-induced peritoneal deterioration after PD-related peritonitis is cured.

## Background

Peritoneal dialysis (PD) is a viable option for renal replacement therapy in patients with end-stage renal disease [1]. Moreover, it is a home-based treatment suitable for improving a patient's quality of life [2]. However, prolonged exposure to dialysis solutions often deteriorates the peritoneum [3]. Peritoneal deterioration is a comprehensive concept that comprises decreased peritoneal function and morphological changes in the peritoneum [4, 5]. Additionally, prolonged exposure to PD fluid triggers chronic inflammation and may contribute to peritoneal deterioration [6]. Therefore, it is important to elucidate the main locus of chronic inflammatory pathogenesis to prevent peritoneal deterioration.

Inflammasomes are intracellular multimeric complex molecules that recognize pathogen-associated or danger-associated molecular patterns [7]. The NLRP3 inflammasome can trigger chronic low-grade inflammation [8] and the pathogenesis of multiple complex diseases, including chronic kidney disease [9], atherosclerosis [10], type 2 diabetes [11], and Alzheimer’s disease [12]. Activation the NLRP3 inflammasome and its downstream pathway particularly reduces pro-caspase-1 activation and caspase-1-mediated interleukine-1β (IL-1β) maturation, accelerating organ fibrosis [13]. Moreover, NLRP3 activation and IL-1β release are critical for solute transport defects and tissue remodeling in PD-associated peritonitis [14]. However, the involvement of inflammasome activation in chronic inflammation after the onset of peritonitis and its role in peritoneal deterioration have not been elucidated. To our knowledge, this study is the first to report persistently high levels of IL-1β in a patient who underwent PD and despite being clinically cured for peritonitis, her peritoneal function decreased in a relatively short period.

## Case presentation

**Fig. 1:**
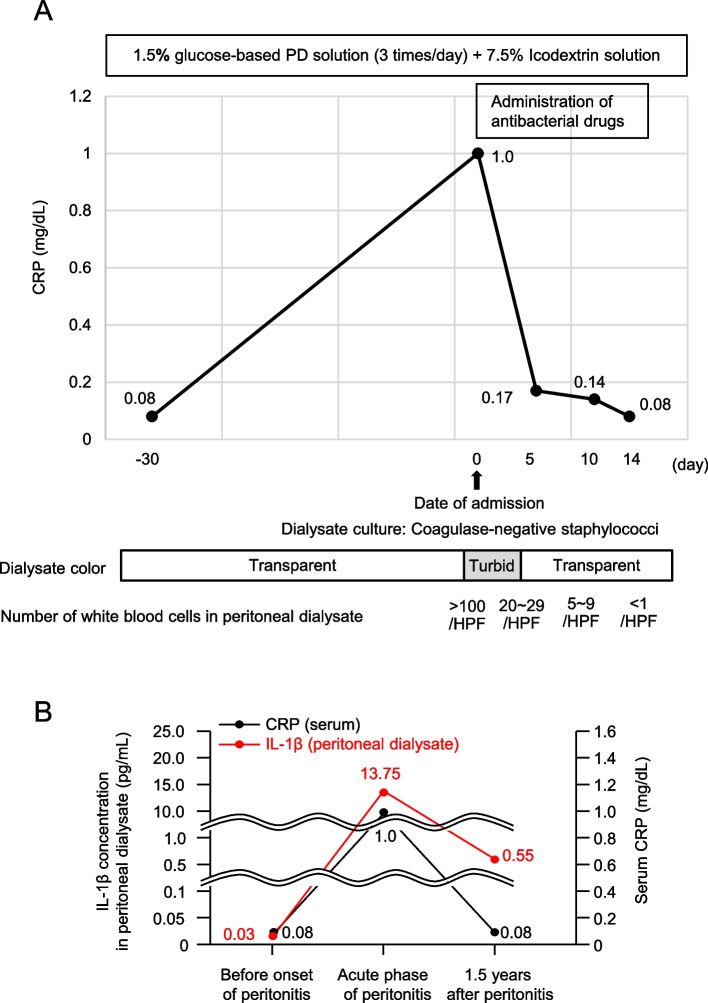
Clinical course and IL-1β concentrations of dialysate. **A** Clinical course during PD-associated peritonitis. After 2 weeks of antimicrobial therapy, peritonitis was clinically cured, and the patient was discharged. **B** Clinical course before and after the onset of bacterial peritonitis. CRP, C-reactive protein; HPF, high-power field; IL-1β, interleukin-1β

**Fig. 2:**
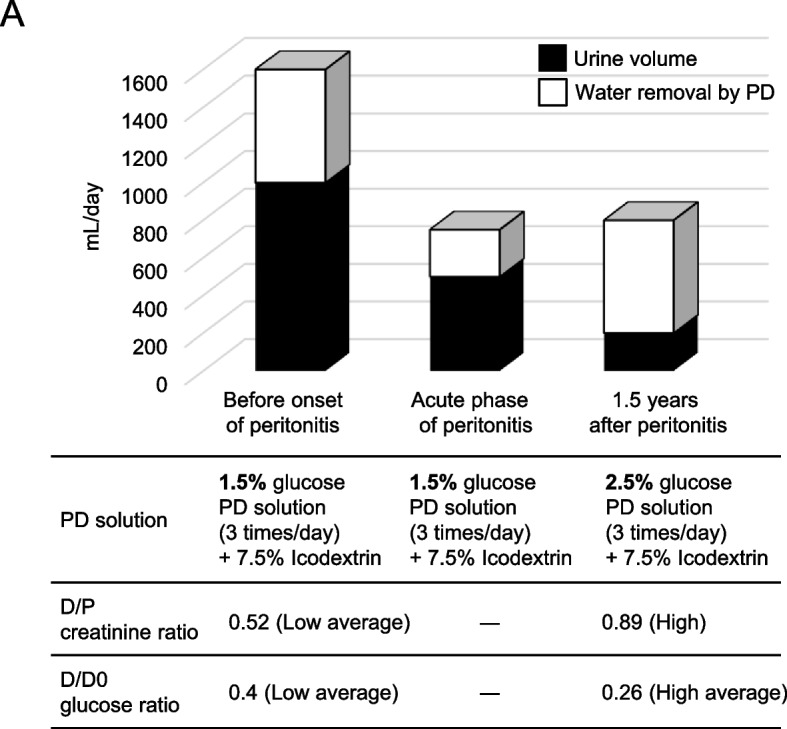
Analysis of peritoneal equilibration test in a patient with PD. **A** Analysis of peritoneal equilibration test before and after the onset of bacterial peritonitis. D, dialysate; P, plasma; PD, peritoneal dialysis

A 64-year-old woman was diagnosed with diabetes mellitus 10 years ago and had been started on PD for end-stage renal disease. Continuous ambulatory PD (low-calcium PD solution with 1.5% dextrose three times per day and 7.5% icodextrin solution once per day; Baxter, Deerfield, IL, USA) was administered, and the patient performed well in an outpatient setting. However, one day, the patient suddenly noticed turbid PD fluid accompanied by a low-grade fever. The patient developed PD-associated acute peritonitis and was admitted to our hospital for treatment. Laboratory results showed elevated C-reactive protein levels and an increased number of white blood cells in the peritoneal dialysate. Coagulase-negative staphylococci were detected in dialysate cultures. Thus, treatment with antimicrobial agents was initiated for PD-associated peritonitis. Dialysate turbidity gradually disappeared after treatment with antimicrobial agents, and the number of cells in the PD fluid decreased. After 2 weeks of antimicrobial therapy, peritonitis was clinically cured, and the patient was discharged (Fig. 1A). Thereafter, the patient did not develop peritonitis; however, residual renal function tended to decline, and peritoneal function also decreased in a relatively short period. We evaluated pro-inflammatory cytokine levels before and after PD-associated peritonitis; interestingly, the levels of IL-1β remained high in the PD fluid, even after remission of bacterial peritonitis (Fig. 1B). However, it became difficult to continue PD any further (Fig. 2A). The protein expression of IL-1β (R&D Systems) in the PD effluent was examined using commercial Quantikine ELISA kits, according to the manufacturer’s protocol. The PD effluent was concentrated using centrifugal filters (Amicon Ultra Centrifugal Filters; Millipore) and the concentration of cytokines in the PD effluent was expressed as pg/mg total protein.

## Discussion and conclusions

To the best of our knowledge, this case report is the first to report an increase in the IL-1β levels of peritoneal dialysate despite healing from PD-related bacterial peritonitis. We also assessed IL-1β expression in the peritoneal dialysate of patients without a history of peritonitis and found no increase in IL-1β levels in the peritoneal dialysate (data not shown), suggesting that IL-1β can affect peritoneal deterioration.

Peritoneal deterioration is inevitable in patients undergoing long-term PD. It is essential to treat patients with PD, while minimizing their peritoneal burden as much as possible. Peritonitis is the most common complication in the aforementioned type of patients [15]. Therefore, preventing it is of utmost importance; if peritonitis develops, surrogate markers are needed to assess subsequent peritoneal deterioration. Recently, NLRP3 inflammasome activation and IL-1β release have been reported to play critical roles in solute transport defects and tissue remodeling in PD-associated acute peritonitis [14]. However, it remains unclear whether inflammasome-dependent inflammatory cytokine levels are persistently elevated after PD-related acute peritonitis. We showed that, even if patients who underwent PD are clinically cured of PD-related acute peritonitis, chronic inflammation may affect the long-term effectiveness of PD.

As for cells releasing IL-1β, PD-related peritonitis has been reported to occur predominantly in resident peritoneal monocytes and macrophages [14], with the former playing a pivotal role in the progression of peritoneal deterioration [16–18]. Macrophages are likely to remain in peritoneal tissue after the onset of PD-related acute peritonitis. Notably, significant changes in the prevalence of macrophage/monocyte populations vary widely in patients undergoing PD depending on the history of peritonitis [19]. Accordingly, macrophage accumulation in peritoneal tissue may reflect chronic peritoneal inflammation after PD-related acute peritonitis. We were unable to identify the source of cells because we were unable to obtain peritoneal tissue from the patient, making this an issue for future consideration.

What are the possible factors that lead to persistent peritoneal inflammation and lead to rapid deterioration of peritoneal function? We consider the CD44-hyaluronan interaction an important factor in the persistence of inflammasome-derived microinflammation, despite the clinical resolution of peritonitis. We recently reported that inflammasome activation in CD44-positive macrophages contributes to peritoneal degradation [20]. Based on our most recent research data, we used in vivo imaging techniques to demonstrate that CD44-positive macrophages infiltrate the subperitoneal mesothelial cells from the blood as a mechanism for the development of peritoneal fibrosis. The ligand for CD44 is hyaluronic acid (HA), which has emerged as an important adhesion molecule for cellular trafficking in multiple organs and contributes to the pathogenesis of various inflammatory diseases [21]. HA in peritoneal dialysate may be useful as a marker for assessing functional and morphological changes in patients undergoing long-term PD [22]. Moreover, CD44 and subsequent HA catabolism trigger the activation of inflammasomes [23]. Although we were not able to analyze this in our case report, it is likely that some mechanism caused small amounts of HA and CD44-positive macrophages to remain after the peritonitis had healed, resulting in persistent inflammation. HA accumulation and several CD44-positive macrophages may regulate the transition from acute to chronic inflammation.

Several studies have indicated that diabetes mellitus (DM) is a risk factor for PD-associated peritonitis [24–26]. Patients with diabetes are compromised hosts and experience several complications. In addition, PD is often mistaken as a potential contributor to visual disorders and peripheral neuropathy [27, 28]. Moreover, Joshi et al. found that glucose load impairs the peritoneal defense system [29]. Thus, DM may affect the incidence of PD-associated peritonitis through several mechanisms.

Regarding the association between DM and decline of residual renal function, inflammasome activation has also been reported in patients with DM [30]. In a previous study, renal biopsies of patients with diabetic nephropathy with proteinuria revealed the expression of inflammasome-associated proteins, such as caspase-1, IL-1β, and IL-18, in the distal and proximal tubules, which is correlated with the degree of proteinuria [31]. Proteinuria can independently predict the rate of decline in residual renal dysfunction in patients with PD [32–34]. Thus, inflammasome activation in diabetic nephropathy may have contributed to the decline in residual renal function in our patient. Conversely, it is difficult to explain why residual renal function declined after peritonitis in our case. It is evident that a multifunctional process, including fluctuations in body fluid status, the antibiotics used for the peritonitis [35], and the peritonitis itself, may individually or collectively contribute to this process. Moreover, the persistence of inflammasome activation after peritonitis may contribute to the decline of residual renal function in patients with DM through some mechanism. However, there are no reports that diabetes itself contributes to peritoneal deterioration associated with inflammasome activation. Furthermore, whether inflammasome-derived proinflammatory cytokines in the peritoneal dialysate of patients with PD contribute to peritoneal degradation remains unclear. Our study could not confirm a causal relationship between IL-1β and peritoneal deterioration. However, in patients without a history of peritonitis, IL-1β levels in the peritoneal dialysate were not elevated, and their peritoneal function was not compromised (data not shown). We consider IL-1β immediately before and after the onset of peritonitis to be clinically essential findings. Increasing the number of cases in future studies to determine whether inflammasome-derived IL-1β serves as a surrogate marker for peritoneal deterioration after peritonitis will have great clinical significance.

In conclusion, the findings of this case report suggest that IL-1β expression in peritoneal dialysate is an important factor in peritoneal deterioration after healing of PD-associated peritonitis. After the onset of bacterial-induced acute peritonitis in patients undergoing PD, inflammasome-dependent inflammation was prolonged, even though peritonitis was clinically cured. Thus, inflammasome-derived pro-inflammatory cytokines may contribute to chronic inflammation-induced peritoneal deterioration after PD-related peritonitis is cured.

## Data Availability

No datasets were generated or analyzed during the current study.
